# Predictors of mortality among TB-HIV Co-infected patients being treated for tuberculosis in Northwest Ethiopia: a retrospective cohort study

**DOI:** 10.1186/1471-2334-13-297

**Published:** 2013-07-01

**Authors:** Balewgizie Sileshi, Negussie Deyessa, Belaineh Girma, Muluken Melese, Pedro Suarez

**Affiliations:** 1 Department of Epidemiology and Biostatistics, College of Health Sciences, Haramaya University, Harar, Ethiopia; 2 Department of Epidemiology and Biostatistics, School of Public health, Addis Ababa University, Addis Ababa, Ethiopia; 3 Help Ethiopia Address Low TB (HEAL TB) Project, USAID/Management Sciences for Health (MSH), Addis Ababa, Ethiopia; 4 Management Sciences for Health (MSH), Arlington, Virginia, USA

**Keywords:** Predictors, Mortality, TB-HIV, Co-infection

## Abstract

**Background:**

Tuberculosis (TB) is the leading cause of mortality in high HIV-prevalence populations. HIV is driving the TB epidemic in many countries, especially those in sub-Saharan Africa. The aim of this study was to assess predictors of mortality among TB-HIV co-infected patients being treated for TB in Northwest Ethiopia.

**Methods:**

An institution-based retrospective cohort study was conducted between April, 2009 and January, 2012. Based on TB, antiretroviral therapy (ART), and pre-ART registration records, TB-HIV co-infected patients were categorized into “On ART” and “Non-ART” cohorts. A Chi-square test and a *T*-test were used to compare categorical and continuous variables between the two groups, respectively. A Kaplan-Meier test was used to estimate the probability of death after TB diagnosis. A log-rank test was used to compare overall mortality between the two groups. A Cox proportional hazard model was used to determine factors associated with death after TB diagnosis.

**Results:**

A total of 422 TB-HIV co-infected patients (i.e., 272 On ART and 150 Non-ART patients) were included for a median of 197 days. The inter-quartile range (IQR) for On ART patients was 140 to 221 days and the IQR for Non-ART patients was 65.5 to 209.5 days. In the Non-ART cohort, more TB-HIV co-infected patients died during TB treatment: 44 (29.3%) Non-ART patients died, as compared to 49 (18%) On ART patients died. Independent predictors of mortality during TB treatment included: receiving ART (Adjusted Hazard Ratio (AHR) =0.35 [0.19-0.64]); not having initiated cotrimoxazole prophylactic therapy (CPT) (AHR = 3.03 [1.58-5.79]); being ambulatory (AHR = 2.10 [1.22-3.62]); CD4 counts category being 0-75cells/micro liter, 75-150cells/micro liter, or 150-250cells/micro liter (AHR = 4.83 [1.98-11.77], 3.57 [1.48-8.61], and 3.07 [1.33-7.07], respectively); and treatment in a hospital (AHR = 2.64 [1.51-4.62]).

**Conclusions:**

Despite the availability of free ART from health institutions in Northwest Ethiopia, mortality was high among TB-HIV co-infected patients, and strongly associated with the absence of ART during TB treatment. In addition cotrimoxazol prophylactic therapy remained important factor in reduction of mortality during TB treatment. The study also noted importance of early ART even at higher CD4 counts.

## Background

The human immunodeficiency virus (HIV) pandemic presents a massive challenge to the control of tuberculosis (TB) at all levels. The synergy between TB and HIV is strong; in high HIV prevalence population, TB is a leading cause of morbidity and mortality, and HIV is driving the TB epidemic in many countries, especially those in sub-Saharan Africa [1]. TB is often the first clinical indication that a person has an underlying HIV infection and, as a result, TB services can be a critical entry point for HIV prevention, care, and treatment [2].

The syndemic interaction between HIV and TB epidemics has had deadly consequences around the world, and disproportionately affects people in Africa [3].

In patients with advanced acquired immune deficiency syndrome (AIDS) and active TB, highly active antiretroviral therapy (HAART) may be administered concurrently with the TB treatment to prevent opportunistic infections which may superimpose and accelerate HIV disease progression [4]. The World Health Organization (WHO) currently recommends that ART should be initiated for all TB-HIV co-infected patients irrespective of their CD4 counts [5].

Despite international recommendations and the proven benefit of ART, physicians remain reluctant to prescribe ART to HIV-infected TB patients, due to concerns about overlapping toxicity, drug-drug interactions, pill burden, and immune reconstitution inflammatory syndrome (IRIS) [6].

Understanding the predictors of mortality for TB-HIV co-infected patients in the local context is critical for Ethiopia to improve TB-HIV co-infected patients’ co-management. To date, there is inadequate data on predictors of mortality among TB-HIV co-infected patients in Ethiopia. To address this, the USAID-funded Help Ethiopia Address Low TB (HEAL TB) project conducted a retrospective study in Northwest Ethiopia to determine predictors of mortality among TB-HIV co-infected patients. The study also aimed to compare the survival rate between TB-HIV co-infected patients who received ART and did not receive ART. It is anticipated that findings from this study will contribute to the body of knowledge that informs TB-HIV program planers, decision makers, and project implementers by providing predictors of mortality among TB-HIV co-infected patients during TB treatment in Ethiopia.

## Methods

### Setting

We conducted a retrospective cohort study in governmental health institutions in Bahir Dar, Northwest Ethiopia, from August, 2011 to January, 2012. Bahir Dar is located in Northwest Ethiopia, 565 kilometers from Addis Ababa. In these health institutions, patients diagnosed as having HIV in any of HIV counseling and testing protocols (i.e., Voluntary counseling and testing, Provider initiated HIV counseling and testing units) are registered in Pre-ART and ART log books according to the status of disease progression. Patients are also referred to ART clinics for pre-ART and ART follow up from private health facilities within Bahir Dar and health facilities outside of Bahir Dar. Felege Hiwot Referral Hospital and Bahir Dar Health Center have provided pre-ART and ART services since 2005 and other health centers in the town began providing these services in 2009. Felege Hiwot Referral Hospital’s 2011 annual report showed that the facility had detected 1,600 TB cases, enrolled 13,590 people living with HIV/AIDS (PLWHA) in ART clinic, and started 9,222 PLWHA on ART. As of 2011 annual report, there were 5,547 PLWHA taking ART at Felege Hiwot Referral Hospital. According to 2011/12 report of Bahir Dar Health Center, a total of 4, 420 PLWHA ever enrolled of which 1, 133 were currently on ART. In the same year the health center reported 135 TB patients. The 2011/12 report of Abay Health Center showed 756 PLWHA enrolled of which 326 were currently on ART. The health center also reported 162 TB patients. Han Health Center reported 1, 726 PLWHA were ever enrolled (407 currently on ART) and 112 TB patients were registered in the year 2011/12.

### Participants

All TB-HIV co-infected patients who started ART before initiating TB treatment, and those who started ART while being treated for TB, were included in the “On ART” cohort. Patients who did not receive ART until completion of TB treatment were included in the “Non-ART” cohort. For both cohorts, inclusion criteria included TB-HIV co-infected patients, aged 15 years or older, who were diagnosed with TB at any time during pre-ART and or ART follow-up since April 2009, and who completed TB treatment before January 2012. Patients who had been diagnosed for both TB and HIV during their initial visit to the health facility were also included for the study.

### Enrollment procedures for study subjects

**Figure 1:**
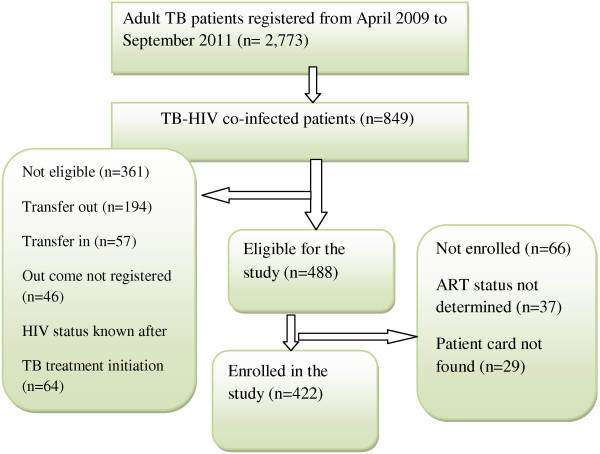
Enrollment of TB-HIV co-infected patients in the study, 2012.

Bahir Dar town was chosen purposely to get adequate number of sample with proper and complete patient record profile. In the town there are seven governmental health institutions, of which three were newly opened during data collection period. Therefore we included four health institutions (Felege Hiwot Refferal Hospital, Bahir Dar Health Center, Han Health Center and Abay Health Center) for the study which delivers TB service, Pre-ART and ART service for TB/HIV co-infected patients. During April 2009 – September 2011, 849 TB-HIV co-infected patients were registered in four health institutions. A total of 422 TB-HIV co-infected patients (272 ‘On ART’ and 150 ‘Non-ART’ cohorts) were included for the study [Figure 1].

### Data collection

Nurses who work in TB and ART clinics were selected to collect data from August, 2011 to January, 2012. Data was collected retrospectively by reviewing the files of TB-HIV co-infected patients in Bahir Dar. All profiles of TB-HIV co-infected patients between April 2009 and January 2012 were considered for data collection. Pre-ART registers, lab requests, follow-up forms, anti-TB record forms, ART intake forms, and patient cards were reviewed. The patients’ date of death was extracted from TB registration log books. Data quality was assured by using a pre-tested data collection tool and trained data collectors. Two public health professionals (Master of public health) had provided continuous supervision and monitoring. Supervisors, data clerks and investigators had checked completeness and consistency of data before and after data entry.

### Measurement of variables

Death from any cause during TB treatment was listed as “on-treatment TB death,” according to the WHO’s TB treatment outcomes definitions [7]. If the date of ART was more than one week before TB treatment initiation, that person was classified as “on ART prior to TB treatment”. Patients who initiated ART at any time before TB treatment was completed were classified as “having received ART during TB treatment”.

Patients were diagnosed with smear positive pulmonary tuberculosis (PTB+), if one of the sputum examinations was positive for acid fast bacilli (AFB). Patients were diagnosed with extra pulmonary tuberculosis (EPTB) if physicians suspected or observed that the TB infection had spread outside of the respiratory organs [5].

Functional status is measured at base line, and a person is categorized into working “able to perform usual work in or out of the house”; Ambulatory “able to perform activities of daily living” and Bedridden “not able to perform activities of daily living”.

### Statistical analysis

Data was entered to EpiData 3.1^a^ for Windows. Statistical package for social science (SPSS) version 16.0 for Windows and Stata version 11.0 were used for analysis. Data was cleaned and edited by simple frequencies and cross tabulation before analysis. The response variable was survival time, defined as “time in days transpired from the date of initial TB treatment to death” or, in the case of individuals who did not die (censored), “the time in days transpired to complete TB treatment”.

Mean (with standard deviation), median (with inter quartile range [IQR]), and frequencies (as percentages) were used to describe patients’ characteristics in each cohort. A Chi-square test and a *T*-test were used to compare categorical and continuous variables between the two cohorts, respectively. The Kaplan-Meier test was used to estimate the probability of death and the median time to death after TB diagnosis. The log-rank test was used to compare time to death between the two groups. The Cox proportional hazard model was used to determine predictors of death after TB diagnosis. All statistically significant (p < 0.05) factors in the bivariate analysis were included in the final model. The crude and adjusted hazard ratio (HR) and its 95% confidence interval (CI) were estimated.

### Ethical issues

Ethical clearance for this study was obtained from the Review Ethics Committee of the School of Public Health at Addis Ababa University. To preserve patient confidentiality, nurses working in the ART clinics extracted the data from patients’ medical records. Moreover, no personal identifiers were used on the data collection form.

## Results

A total of 422 TB-HIV co-infected patients (272 On ART patients and 150 Non-ART patients) were included for the study and followed for a median of 197 days with an IQR of 140 to 221 days among On ART patients and 191 days among Non-ART patients with an IQR of 65.5 to 209.5 days.

### Baseline socio-demographic characteristics of the study subjects

In this study, the two cohorts were not statistically different in any of the identified socio-demographic attributes. The median age of study subjects in both cohorts was 30 years with an IQR of 27 to 37.5 years in the On ART cohort and 25 to 38 years in the Non-ART cohort. There were more female than male subjects in both cohorts, with 141 (53.4%) women in the On ART cohort and 83 (56.5%) women in the Non-ART cohort. More than one third of patients in both cohorts had completed secondary school with 93 (34.8%) in the On ART cohort and 50 (35.7%) in the Non-ART cohort (see Table 1).

**Table 1:** Baseline socio-demographic characteristics of TB-HIV co- infected patients in Bahir Dar town, 2012

**Base line variable**	**On ART (n = 272)**	**Non-ART (n = 150)**	***X*2 Value (df)**	**P-Value**
**Residency (n = 407)**				
Urban	235 (90.7%)	129 (87.2%)	1.271	0.260
Rural	24 (9.3%)	19 (12.8%)	(1)	
**Age (n = 408)**				
Mean ± SD	32.58 ± 9.123	31.98 ± 9.837	0.753♦	0.452
Median (IQR)	30 yrs (27-37.5)	30 yrs (25-38)		
**Sex (n = 411)**				
Male	123 (46.6%)	64 (43.5%)	0.355	0.551
Female	141 (53.4%)	83 (56.5%)	(1)	
**Religion (n = 411)**				
Orthodox	226 (85.3%)	117 (80.2%)	5.962	0.183
Muslim	26 (9.8%)	18 (12.3%)	(2)	
Others	13 (4.9%)	11 (7.5%)		
**Marital status (n = 415)**				
Single	76 (28.1%)	55 (37.9%)	7.901	0.131
Married	108 (40.0%)	57 (39.3%)	(3)	
Divorced	52 (19.2%)	24 (16.6%)		
Widowed	34 (12.6%)	9 (6.2%)		
**Educational status (n = 407)**				
Not educated	77 (28.8%)	40 (28.6%)	0.765	0.858
Primary	61 (22.8%)	35 (25.0%)	(3)	
Secondary	93 (34.8%)	50 (35.7%)		
Tertiary	36 (13.5%)	15 (10.7%)		

♦ = *T*-test Statistic for independent sample test used.

### Clinical characteristics of the study subjects

The clinical condition of study subjects within the two cohorts was not statistically different among any of the identified variables, except for history of prophylactic medication. In the On ART cohort, a higher proportion (51.6%) of patients had used prophylactic medication, as compared to patients in the Non-ART cohort (27.1%), (*X*^2^ = 21.721; df (1); p = 0.000). Among all study subjects, more than one third had had at least one past opportunistic infection. In the On ART cohort, 58 (22.4%) study subjects had a history of past TB treatment, as compared to just 31 (21.7%) in the Non-ART cohorts. Data showed that the On ART and Non-ART cohorts had statistically different median CD4 counts (T = 10.305; p = 0.000): the On ART cohort had a much lower CD4 count with, a median of 114 cells/micro liter (μl) and an IQR of 58 to 185 cells/μl, as compared to the Non-ART cohort, which had a median of 291 cells/μl and an IQR of 183.5 to 448 cells/μl (see Table 2).

**Table 2:** Baseline clinical characteristics of TB-HIV co- infected patients in Bahir Dar town, 2012

**Base line variable**	**On ART (n = 272)**	**Non-ART (n = 150)**	***X***^**2**^**Value (df)**	**P- Value**
**Past OIs (n = 366)**				
Yes	105 (43.6%)	55 (44.0%)	0.006	0.937
No	136 (56.4%)	70 (56.0%)	(1)	
**Past TB Treatment (n = 402)**				
Yes	58 (22.4%)	31 (21.7%)	0.027	0.869
No	201 (77.6%)	112 (78.3%)	(1)	
**Functional status (n = 397)**				
Working	159 (60.9%)	80 (58.8%)	1.363	0.506
Ambulatory	72 (27.6%)	44 (32.4%)	(2)	
Bedridden	30 (11.5%)	12 (8.8%)		
**CD4 count (n = 408)**				
Mean ± SD	132.9 ± 94.42	312.78 ± 192.8	10.30♦	0.000*
Median (IQR)	114 (58-185)	291 (183.5-448)		
**Hgb level (mmHg)(n = 357)**				
Mean ± SD	11.36 ± 2.3	11.46 ± 1.83	0.063♦	0.95
Median (IQR)	11.3 (10.0-13.0)	12.0 (10.12-13.0)		
**TB diagnosis**				
Smear Positive PTB	53 (19.5%)	40 (26.7%)	10.434	0.005*
Smear Negative PTB	107 (39.3%)	36 (24.0%)	(2)	
Extra PTB	112 (41.2%)	74 (49.3%)		
**CPT (n = 397)**				
Prescribed	239 (93.0%)	109 (77.9%)	19.19	0.000*
Not Prescribed	18 (7.0%)	31 (22.1%)	(1)	
**Outcome of TB Treatment (n = 417)**				
Cure	37 (13.7%)	16 (11.0%)	8.039	0.045*
Treatment completed	167 (61.6%)	77 (52.7%)	(3)	
Defaulter	18 (6.6%)	9 (6.2%)		
Death	49 (18.1%)	44 (30.1%)		

* Significant at α = 0.05, ♦ = *T*-test Statistic for independent sample test used.

There was a statistically significant difference in the type of TB diagnosis between the cohorts. In the On ART group, 107 (39.3%) study subjects had smear negative PTB, whereas only 36 (24.0%) had smear negative PTB in the Non-ART group (*X*^2^ =10.434; df = 2; p = 0.005). A higher proportion (93.3%) of study subjects in the On ART cohort had received CPT, as compared to those in the Non-ART cohort (77%) (see Table 2).

### Comparison of mortality between the on ART and Non-ART cohorts

**Figure 2:**
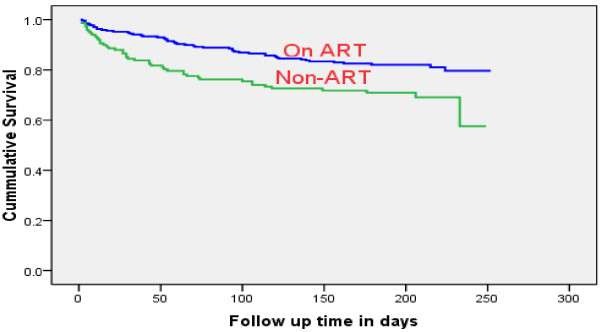
Kaplan-Meier estimate of survival among TB-HIV co-infected patients in Bahir Dar town, 2012.

The 422 study subjects contributed a cumulative total of 2,274.4 person month observations (PMO) to this study; the On ART cohort contributed 1,545.03 PMO and the Non-ART cohort contributed 729.37 PMO. In the Non-ART cohort, 44 (29.3%) of TB-HIV co-infected patients died during TB treatment, which represented a higher percentage than the 49 patients (18%) who died in the On ART cohort. The incidence rate of mortality in the Non-ART cohort was 6.03 per 100 person months observations (PMO), (95% CI: 4.5, 8.1) and the mortality incidence in the On-ART cohort was 3.2 per 100 PMO (95% CI: 2.40, 4.20). The overall incidence rate of mortality during TB treatment was 4.09 per 100 PMO (95% CI: 3.34, 5.01). Results from the On ART cohort showed that incidence of mortality in the first month of TB treatment was 5.4 per 100 PMO and, in the second month of TB treatment, was 4.8 per 100 PMO. The corresponding values in Non-ART cohort was 16.9 per 100 PMO and 5.9 per 100 PMO in the first and second months of TB treatment, respectively. The median time to death was 59 days in the On ART cohort and 29.5 days in the Non-ART cohort. The overall probability of survival in the On ART cohort was significantly greater than in the Non-ART cohort (log rank statistic = 8.93, df = 1, P = 0.003); (see Figure 2).

### Predictors of mortality in TB-HIV Co-infected patients during TB treatment

The bivariate analysis showed that the risk of death decreased by 46% (HR = 0.54, 95% CI: 0.36-0.82) in the On-ART cohort. Compared to smear negative PTB patients, smear positive PTB patients had a 2.02 (95% CI: 1.07-3.83) times higher risk of death and EPTB patients had a 2.77 (95% CI: 1.61-4.75) times higher risk of death. In addition, patients who did not start CPT had a 3.15 times higher risk of mortality (95% CI: 1.95-5.11). Compared to the reference group, TB patients 45 years old or more (HR = 2.58, 95% CI: 1.34-4.92), patients with ambulatory and bedridden functional status (HR = 2.76, 95% CI: 1.71-4.47 and HR = 3.88, 95% CI: 2.15-7.02 respectively), and patients with CD4 count less than 75 cells/μl (HR = 2.08, 95% CI: 1.17- 3.70) had an increased risk of mortality during TB treatment. In the study, completing primary school -reduced risk of death by 55% (HR = 0.45, 95% CI: 0.22-0.90), compared to not educated TB-HIV co-infected patients (see Table 3).

**Table 3:** Predictors of mortality among TB-HIV co-infected patients in Bahir Dar town, 2012

**Variable**	**Number at risk**	**Number of death**	**Incidence of mortality per 100 person month observation (95% CI)**	**Crude hazard ratio (95% CI)**
**ART**				
Started	272	49	3.17 (2.39, 4.19)	0.54 (0.36, 0.82)*
Not started	150	44	6.03 (4.49, 8.11)	1
**CPT prophylaxis**				
Prescribed	348	60	3.59 (2.66, 4.87)	1
Not prescribed	49	23	10.18 (6.77, 15.32)	3.15 (1.95, 5.11)*
**Type of TB**				
Smear negative PTB	143	17	2.02 (1.26, 3.25)	1
Smear positive PTB	93	21	4.31 (2.81, 6.61)	2.02 (1.07, 3.83)*
Extra PTB	186	55	5.81 (4.46, 7.57)	2.77 (1.61, 4.78)*
**Past OIs**				
No	206	50	4.62 (3.50, 6.09)	1
Yes	160	31	3.53 (2.48, 5.02)	0.77 (0.49, 1.20)
**Past TB treatment**				
No	313	66	3.95 (3.10, 5.03)	1
Yes	89	18	3.58 (2.25, 5.68)	0.91 (0.54, 1.54)
**Functional status**				
Working	239	31	2.21 (1.55, 3.14)	1
Ambulatory	116	36	6.39 (4.61, 8.87)	2.77 (1.71, 4.47)*
Bedridden	42	17	7.31 (3.81, 14.05)	3.88 (2.15, 7.02)*
**CD4 count**				
< 75	107	33	6.22 (4.42, 8.75)	2.08 (1.17, 3.30)*
75-150	91	21	4.44 (2.90, 6.81)	1.50 (0.80, 2.82)
150-250	95	19	3.57 (2.28, 5.59)	1.24 (0.65, 2.37)
> = 250	115	18	2.89 (1.82, 4.59)	1
**Health institution**				
Health center	226	34	2.67 (1.91, 3.72)	1
Hospital	196	59	5.89(4.57, 7.61)	2.18 (1.43, 3.33)*
**Age(n = 408)**				
15-24	100	18	3.30 (2.08, 5.24)	1
25-34	193	40	3.79 (2.78, 5.17)	1.15 (0.66, 2.01)
35-44	69	16	4.40 (2.70, 7.18)	1.30 (0.66, 2.55)
> = 45	46	19	8.97 (5.72, 14.06)	2.58 (1.34, 4.92)*
**Sex**				
Male	187	45	4.53 (3.36, 6.07)	1
Female	224	45	3.69 (2.75, 4.94)	0.82 (0.54, 1.24)
**Educational status**				
Not educated	117	26	4.47 (3.05, 6.57)	1
Primary	96	11	1.93 (1.07, 3.49)	0.45 (0.22, 0.90)*
Secondary	143	37	4.82 (3.49, 6.66)	1.10 (0.66, 1.81)
Tertiary	51	13	4.48 (2.60, 7.72)	1.05 (0.54, 2.05)

*Significant at α = 0.05.

ART status, CPT status, CD4 count, functional status, type of TB diagnosis, and type of health institution were independent predictors of mortality after controlling for the other factors. From these factors, receiving ART during TB treatment had decreased risk of mortality by 65% (AHR = 0.35, 95% CI: 0.19-0.64). In addition, CPT remained an important factor in reduction of mortality during TB treatment, in which patients without CPT were at a 3.03 times higher risk of mortality (95% CI: 1.58, 5.79). In this study CD4 count categories 0-75 cells/μl, 75-150 cells/μl, and 150-250 cells/μl; EPTB type; being ambulatory; and treatment in a hospital were independent predictors of increased risk of mortality during TB treatment (see Table 4).

**Table 4:** Multivariate predictors of mortality among TB-HIV co-infected patients in Bahir Dar town, 2012

**Variable**	**Crude hazard ratio (95% CI)**	**Adjusted hazard ratio (95% CI)**
**ART**		
Started	0.54 (0.36, 0.82)	0.35 (0.19, 0.64)*
Not started	1	1
**CPT prophylaxis**		
Prescribed	1	1
Not prescribed	3.15 (1.95, 5.11)	3.03 (1.58, 5.79)*
**Type of TB**		
Smear negative PTB	1	1
Smear positive PTB	2.02 (1.07, 3.83)	2.11 (0.95, 4.65)
Extra PTB	2.77 (1.61, 4.78)	2.39 (1.23, 4.66)*
**CD4 count**		
< 75	2.08 (1.17, 3.30)	4.83 (1.98, 11.78)*
75-150	1.50 (0.80, 2.82)	3.57 (1.48, 8.61)*
150-250	1.24 (0.65, 2.37)	3.07 (1.33, 7.07)*
> = 250	1	1
**Functional status**		
Working	1	1
Ambulatory	2.77 (1.71, 4.47)	2.10 (1.22, 3.62)*
Bedridden	3.88 (2.15, 7.02)	2.11 (0.98, 4.53)
**Health Institution**		
Health center	1	1
Hospital	2.18 (1.43, 3.33)	2.64 (1.51, 4.62)*
**Age(n = 408)**		
15-24	1	1
25-34	1.15 (0.66, 2.01)	1.17 (0.60, 2.29)
35-44	1.30 (0.66, 2.55)	0.98 (0.43, 2.23)
> = 45	2.58 (1.34, 4.92)	2.20 (0.97, 4.59)
**Educational status**		
Not educated	1	1
Primary	0.45 (0.22, 0.90)	0.49 (0.22, 1.12)
Secondary	1.10 (0.66, 1.81)	0.88 (0.49, 1.58)
Tertiary	1.05 (0.54, 2.05)	0.65 (0.29, 1.47)

*Significant at α = 0.05. ART status, health institution type, age, educational status, functional status, CD4 count, type of TB diagnosis, and CPT initiation were included in the model.

## Discussion

This study revealed the overwhelming problem of the high mortality of TB-HIV co-infected patients during TB treatment. More than 1 in 5 TB-HIV co-infected individuals died during TB treatment. Results from this study demonstrated that ART remained independently protective against mortality during TB treatment. In addition not having initiated cotrimoxazole prophylactic therapy; being ambulatory; CD4 count and treatment in a hospital were independent predictors of mortality during TB treatment.

In our study, the median CD4 count in the Non-ART cohort was twice as high as the median CD4 count in the On ART cohort. Non-ART cohorts may have been diagnosed as having HIV and TB, before their clinical and immunological conditions deteriorate. The median CD4 count among participants in this study was much higher than the median CD4 count among participants in other studies [8-15]. The difference may be due to the fact that researchers in our study took CD4 counts while the study subjects were being treated for TB or one month before they began TB treatment and, in most cases, these study subjects had started ART before TB diagnosis, which may have improved their immunological status. In addition, the results showed that 86.3% of study subjects had a CD4 count below 350 cells/μl. This is similar with a study conducted in Zimbabwe where 84.6% of study participants had a CD4 count below 350 cells/μl [16]. This showed that most study subjects were in progressive immunodeficiency condition.

There was no statistical difference in type of TB diagnosis between the two cohorts; 55.9% of study subjects were diagnosed with PTB and 44.1% were diagnosed with EPTB. This is in line with other studies [11,13,17] but the proportion of EPTB in this study is high compared to two studies conducted in India (22.9% and 31%) and one study conducted done in Thailand, which reported that 31% of study subjects had EPTB [10,12,18]. The variation could be a result of stage of HIV disease, difference in TB diagnosis or epidemiology of TB in different countries.

We found that mortality rate was high (22%) among TB-HIV co-infected patients during TB treatment. In line with this, previous studies have reported high mortality rates ranging from 8.5% to 30% among TB-HIV co-infected patients prior to successful completion of TB treatment [4,8-10,12,13,15,17-19]. In our study, death occurred in 49 of 272 patients (18%) exposed to ART during TB treatment, compared with 44 of 150 patients (29.3%) never exposed to ART. This finding is similar to a study conducted in India, where death occurred in 11.3% of patients exposed to ART during TB treatment and 24.6% of TB patients never exposed to ART [10]. However, results from a study conducted in Thailand showed 46% proportion of death among TB-HIV co-infected patients who did not start ART [13]. Another study in Thailand reported that 5 of 71 patients (7%) who received ART died, compared with 94 of 219 patients (43%) who did not receive ART (RR 0.2; 95% CI: 0.1–0.4) [18]. In Malawi, a total of 132 of 660 patients (20%) died during an eight-month course of anti-TBs treatment, which is consistent with our finding of 22% [20].

In this study, we have documented that the risk of mortality was high among subjects in the first month of TB treatment. This may be due to delayed presentation of patients and, thus, advanced TB and HIV/AIDS, late diagnosis of TB within health institutions, and the presence of life-threatening HIV related complications. These results are similar to results from a study conducted in Thailand which showed the first month of TB treatment is the time of the maximum number of deaths [21]. Another study conducted in sub-Saharan Africa concluded that ART should be started soon after TB diagnosis because the majority of deaths among TB-HIV patients in this study occurred during the patients’ first two months of TB treatment [22].

In our retrospective, institution-based study, we found that TB-HIV co-infected patients who took ART during TB treatment had a lower risk of death. This is consistent with studies from several other settings [8-11,13,15,17-19,23,24] that demonstrate the positive impact of ART on the survival outcomes among TB-HIV co-infected patients, including successful immune restoration and reductions in morbidity and mortality.

In addition to ART, we found other immunological factors associated with mortality. For example, mortality rates increased in TB-HIV co-infected patients with lower CD4 counts. This finding is consistent with a study in Zimbabwe, which showed that HIV-TB co-inflected patients with a CD4 count of <50 cells/micro litter had a 13 percent increased risk of death compared to patients with CD4 count greater or equal to 200 cells/micro litter [16]. Oppositely, a study conducted in southern India showed that a CD4 count below 200/mm3 was not associated with a higher rate of mortality [17]. The difference between our results and these others may be that we categorized CD4 counts into smaller intervals, which better enabled us to see the effect of CD4 counts on mortality.

The risk of death during TB treatment was higher in patients treated at a hospital compared to those treated at a health center. The reason could be that those who are taking care in hospitals might have advanced disease conditions. As a result, the severely ill hospitalized patients appeared to have a greater incidence of mortality, as compared to the less ill health center patients.

In this study TB-HIV co-infected patients with extra PTB were at increased risk of mortality during TB treatment compared to smear negative PTB patients. In other studies PTB is associated with high risk of mortality [10]. The possible reason may be HIV infected patients with extra PTB were highly immune-compromised.

In our study not initiating CPT was associated with high risk of mortality. In line with this, studies from South India and Sub-Saharan Africa showed that not taking CPT was significantly associated with mortality [17,22]. In our study, however, patients who died shortly after being diagnosed with TB and HIV may not have had the chance to initiate CPT. This may have led us to overestimate the benefit of CPT.

Our study was subject to several important limitations. All TB-HIV co-infected patients who started ART before initiating TB treatment, and those who started ART while being treated for TB, were included in the same group which may introduce bias. Information about other biomedical predictors for death that may have confounded this study, such as drug resistance, severity of immune suppression, or co-morbidities, adherence of medication were not available. We were also unable to collect adequate information about specific types of EPTB and patients’ recent CD4 counts. Since most deaths in Ethiopia occur at home [25], it was difficult to trace all deaths. Exclusion of patients who transferred out of care may have also slightly confounded our results.

## Conclusions

A significant difference was observed in the mortality rate during TB treatment between the On ART and Non-ART cohorts. Despite the fact that ART is available in most governmental health institutions throughout Ethiopia, death was strongly associated with the absence of ART during TB treatment. Risk of death was 65% lower in TB-HIV co-infected patients treated with ART, as compared to those not treated with ART. In addition cotrimoxazol prophylactic therapy remained important factor in reduction of mortality during TB treatment. The study also noted importance of early ART even at higher CD4 counts. To alleviate this, expanding ART use among TB-HIV co-infected patients is critical to improving the survival of these patients. Health institutions in Ethiopia should begin treating all TB-HIV co‒infected patients with ART, irrespective of CD4count levels, as per the WHO recommendation.

## Endnotes

^a^ EpiData Association, Odense, Denmark.

## Abbreviations

AFB: Acid fast bacilli; AHR: Adjusted hazard ratio; AIDS: Acquired immune deficiency syndrome; ALT: Alanine transaminase; AST: Aspartate aminotransferase; ART: Antiretroviral therapy; CD4: Cluster of differentiation 4; CI: Confidence interval; CPT: Cotrimoxazole prophylactic therapy; Df: Degree of freedom; EPTB: Extra pulmonary tuberculosis; HAART: Highly active antiretroviral therapy; HEAL TB: Help Ethiopia Address Low Tuberculosis (project); HIV: Human immunodeficiency virus; HR: Hazard ratio; IQR: Inter-quartile range; IRIS: Immune reconstitution inflammatory syndrome; MSH: Management Sciences for Health; OR: Odds ratio; PLWHA: People living with HIV and AIDS; PMO: Person months observed; PTB: Pulmonary tuberculosis; PTB+: Smear-positive pulmonary tuberculosis; RR: Relative risk; TB: Tuberculosis; USAID: United States Agency for International Development; WHO: World Health Organization; μl: Micro liter.

## Competing interests

The authors declare that they have no competing interests.

## Authors’ contributions

BS designed the study, performed statistical analysis, and drafted the manuscript. ND participated in the study design and analysis. BG participated in the study design, analysis, and helped to draft the manuscript. MM and PS participated in the study design and helped to draft the manuscript. All of these authors provided critical comments for revision and approved the final version of the manuscript.

## Authors’ information

BS, (MPH), lecturer at Haramaya University; ND (MD, MPH, PhD), Assistant Professor of Psychiatric Epidemiology in Addis Ababa University; BG (MD, MPH), Monitoring and Evaluation Advisor, HEAL TB Project, Ethiopia; MM (MD, MPH), Program Director, HEAL TB Project, Ethiopia; PS (MD,), Director of TB and TB-HIV, Management Science for Health, USA.

## Pre-publication history

The pre-publication history for this paper can be accessed here:

http://www.biomedcentral.com/1471-2334/13/297/prepub

## References

[B1] Federal Minister of Health Implementation Guideline for TB/HIV Collaborative Activities in Ethiopia 2007 Addis Ababa: Federal Ministry of Health

[B2] World Health Organization Interim policy on collaborative TB/HIV activities 2004 Geneva: World Health Organization

[B3] Kwan CK Ernst JD HIV and Tuberculosis: a deadly human syndemic Clin Microbiol Rev 2011 24 2 351 376 Apr 10.1128/CMR.00042-10 21482729 PMC3122491

[B4] Dean GL Edwards SG Ives NJ Matthews G Fox EF Navarante L Fisher M Taylor GP Miller R Taylor CB de Ruiter A Pozniak AL Treatment of tuberculosis in HIV-infected persons in the era of highly active antiretroviral therapy AIDS 2002 16 75 83 10.1097/00002030-200201040-00010 11741165

[B5] World Health Organization Treatment of tuberculosis guidelines 2010 4 Geneva: World Health Organization 23741786

[B6] Burman WJ Issues in the management of HIV related tuberculosis Clin Chest Med 2005 2 283 294 15837111 10.1016/j.ccm.2005.02.002

[B7] World Health Organization Treatment of Tuberculosis: Guidelines for National Programmes 2003 3 Geneva: World Health Organization

[B8] Cain KP Anekthananon T Burapat C Akksilp S Mankhatitham W Srinak C Nateniyom S Sattayawuthipong W Tasaneeyapan T Varma JK Causes of death in HIV-infected persons who have tuberculosis, Thailand Emerg Infect Dis 2009 15 2 258 264 February 10.3201/eid1502.080942 19193270 PMC2657626

[B9] Gadkowski LB Hamilton CD Allen M Fortenberry ER Luffman J Zeringue E Stout JE HIV-specific health care utilization and mortality among Tuberculosis/HIV coinfected persons AIDS Patient Care STDS 2009 23 10 845 851 October 10.1089/apc.2009.0030 19803793 PMC2832656

[B10] Raizada N Chauhan LS Babu BS Thakur R Khera A Wares DF Sahu S Bachani D Rewari BB Dewan PK Linking HIV-infected TB patients to cotrimoxazole prophylaxis and antiretroviral treatment in India PLoS One 2009 4 6 e5999 10.1371/journal.pone.0005999 19543396 PMC2695556

[B11] Manosuthi W Chottanapand S Thongyen S Chaovavanich A Sungkanuparph S Survival rate and risk factors of mortality among TB/HIV co-infected patients with and without antiretroviral therapy Acquir Immune Defic Syndr 2006 43 1 42 46 September 10.1097/01.qai.0000230521.86964.86 16885778

[B12] Varma JK Nateniyom S Akksilp S Mankatittham W Sirinak C Sattayawuthipong W Burapat C Kittikraisak W Monkongdee P Cain KP Wells CD Tappero JW HIV care and treatment factors associated with improved survival during TB treatment in Thailand: an observational study BMC Infect Dis 2009 42 9 April 10.1186/1471-2334-9-42 PMC2674442 19364398

[B13] Sanguanwongse N Cain KP Suriya P Nateniyom S Yamada N Wattanaamornkiat W Sumnapan S Sattayawuthipong W Kaewsa-ard S IngKaseth S Varma JK Antiretroviral therapy for HIV-infected tuberculosis patients saves lives but needs to be used more frequently in Thailand J Acquir Immune Defic Syndr 2008 48 181 189 10.1097/QAI.0b013e318177594e 18520676

[B14] Umphonsathien M Sungkanuparph S Early initiation of antiretroviral therapy in HIV/Tuberculosis co-infection and immune reconstitution inflammatory syndrome J Infect Dis Antimicrob Agents 2011 28 15 23

[B15] Worodria W Massinga-Loembe M Mazakpwe D Luzinda K Menten J Van Leth F Kizza HM Kestens L Mugerwa RD Reiss P Colebunders R Incidence and predictors of mortality and the effect of tuberculosis immune reconstitution inflammatory syndrome in a cohort of TB/HIV patients commencing antiretroviral therapy J Acquir Immune Defic Syndr 2011 58 1 32 37 10.1097/QAI.0b013e3182255dc2 21654499

[B16] MacPherson P Dimairo M Bandason T Zezai A Munyati SS Butterworth AE Mungofa S Rusakaniko S Fielding K Mason PR Corbett EL Risk factors for mortality in smear-negative tuberculosis suspects: a cohort study in Harare, Zimbabwe Int J Tuberc Lung Dis 2011 15 10 1390 1396 10.5588/ijtld.11.0056 22283900 PMC3272461

[B17] Vijay S Kumar P Chauhan LS Narayan Rao SV Vaidyanathan P Treatment outcome and mortality at one and half year follow-Up of HIV infected TB patients under TB control programme in a District of South India PLoS One 2011 6 7 e21008 10.1371/journal.pone.0021008 21814542 PMC3144198

[B18] Akksilp S Karnkawinpong O Wattanaamornkiat W Viriyakitja D Monkongdee P Sitti W Rienthong D Siraprapasiri T Wells CD Tappero JW Varma JK Antiretroviral therapy during tuberculosis treatment and marked reduction in death rate of HIV infected patients, Thailand Emerg Infect Dis 2007 13 7 1001 1007 10.3201/eid1307.061506 18214171 PMC2878231

[B19] Straetemans M Glaziou P Bierrenbach AL Si Smanidis C Van der Werf MJ Assessing tuberculosis case fatality ratio: a meta-analysis PLoS ONE 2011 6 6 e20755J 21738585 10.1371/journal.pone.0020755 PMC3124477

[B20] Zachariah R Fitzgerald M Massaquoi M Acabu A Chilomo D Salaniponi FML Harries AD Does antiretroviral treatment reduce case fatality among HIV-positive patients with tuberculosis in Malawi? Int J Tuberc Lung Dis 2007 11 8 848 853 17705949

[B21] Moolphate S Aung MN Nampaisan O Nedsuwan S Kantipong P Suriyon N Hansudewechakul C Yanai H Yamada N Ishikawa N Time of highest tuberculosis death risk and associated factors: an observation of 12 years in northern Thailand Int J Gen Med 2011 4 181 190 February 21475634 10.2147/IJGM.S16486 PMC3068883

[B22] Harries AD Zachariah R Lawn SD Providing HIV care for co-infected tuberculosis patients: a perspective from sub-Saharan Africa Int J Tuberc Lung Dis 2009 13 6 16 19105873

[B23] Nahid P Gonzalez LC Rudoy I de Jong BC Unger A Kawamura LM Osmond DH Hopewell PC Daley CL Treatment outcomes of patients with HIV and tuberculosis Am J Respir Crit Care Med 2003 167 603 17290042 10.1164/rccm.200509-1529OC PMC1899273

[B24] Franke MF Robins JM Mugabo J Kaigamba F Cain LE Fleming JG Murray MB Effectiveness of early antiretroviral therapy initiation to improve survival among HIV infected adults with tuberculosis: a retrospective cohort study PLoS Med 2011 8 5 e1001029 10.1371/journal.pmed.1001029 21559327 PMC3086874

[B25] Lulu K Berhane Y The use of simplified verbal autopsy in identifying causes of adult death in a predominantly rural population in Ethiopia BMC Publ Health 2005 5 58 10.1186/1471-2458-5-58 PMC1164421 15935096

